# Mechanism of HIF-1α-mediated angiogenesis in rheumatoid arthritis and progress of natural medicine interventions

**DOI:** 10.3389/fphar.2026.1793851

**Published:** 2026-07-03

**Authors:** Yingjun Wei, Xingwen Xie, Xuan Hou, Dingpeng Li, Junhu Hou, Yaxiong Gao

**Affiliations:** 1 Gansu University of Chinese Medicine, Lanzhou, China; 2 Gansu Provincial Hospital of Traditional Chinese Medicine, Lanzhou, China; 3 The First Affiliated Hospital of Gansu University of Chinese Medicine, Lanzhou, China; 4 Gansu Medical College, Pingliang, China; 5 Affiliated Hospital of Gansu University of Chinese Medicine, Lanzhou, China

**Keywords:** angiogenesis, HIF-1α, natural medicine, rheumatoid arthritis, signaling pathway

## Abstract

Rheumatoid arthritis (RA) is a chronic autoimmune disease characterized by hyperplasia of synovial pannus and progressive joint destruction. Pathological angiogenesis, driven by hypoxia-inducible factor-1α (HIF-1α), constitutes a core pathological mechanism and has become a critical therapeutic target for RA. This article systematically elucidates the molecular mechanisms by which HIF-1α drives pathological angiogenesis through interactions with signaling pathways such as vascular endothelial growth factor (VEGF), angiogenin (ANG)-1/2, CXC chemokine ligand 12 (CXCL12)/CXC chemokine receptor 4 (CXCR4), phosphatidylinositol 3-kinase (PI3K)/protein kinase B (Akt)/mammalian target of rapamycin (mTOR). HIF-1α-mediated angiogenesis forms a positive feedback network with pathological processes including synovial inflammatory response, glycolytic metabolic reprogramming, and oxidative stress-mitochondrial damage, collectively promoting synovial pannus formation. Additionally, this article summarizes the effects of traditional Chinese medicine formulations and plant-derived monomeric metabolites on HIF-1α-mediated RA synovial angiogenesis, as well as the preclinical research advances of anti-angiogenic mechanisms. It also briefly explores the potential of novel botanical drugs delivery systems in enhancing the targeting of natural products to the HIF-1α pathway and improving therapeutic efficacy, aiming to provide new perspectives and strategic options for HIF-1α-targeted RA therapies.

## 1 Introduction

Rheumatoid arthritis (RA) is a chronic autoimmune disease pathologically characterized by persistent synovitis, proliferative synovial pannus formation, and progressive cartilage and bone destruction (Díaz-González and Hernández-Hernández, 2023). Epidemiological studies indicate an increasing prevalence of RA since 1990 (Radu and Bungau, 2021). Angiogenesis is one of the key pathological features in the early stages of RA (Elshabrawy et al., 2015). Angiogenesis can promote the proliferation and activation of fibroblast-like synovial cells (FLS) and facilitate pannus formation, leading to sustained disease progression, triggering bone destruction, and ultimately resulting in joint deformity and dysfunction (Maeda et al., 2022). Concurrently, the formation of a hypoxic microenvironment in RA synovial tissue (Zhu et al., 2019; Sparks, 2019; Chen J. et al., 2023) is one of the key driving factors of synovial angiogenesis (Gong et al., 2019). Therefore, inhibiting synovial angiogenesis in the early stages of RA is crucial for its treatment (Eelen et al., 2020).

**FIGURE 1:**
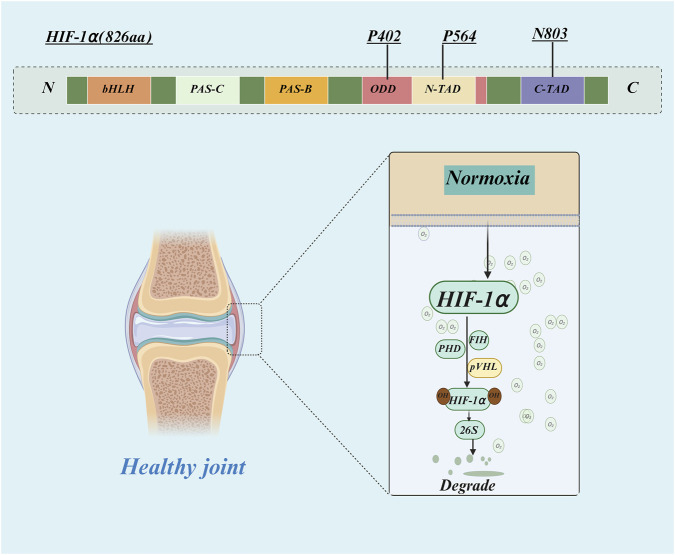
Structure of HIF-1α and its degradation under normoxic conditions [Created with BioRender (https://app.biorender.com/)].

Hypoxia-inducible factor (HIF)-1α is an oxygen-sensitive transcriptional regulator that coordinates cellular adaptation to hypoxic and ischemic conditions (Zhang et al., 2014; Muz et al., 2012; Acuña-Pilarte and Koh, 2025). By sensing fluctuations in oxygen tension and transactivating a broad array of downstream pro-angiogenic genes, HIF-1α not only directly drives the transcription of key pro-angiogenic factors such as vascular endothelial growth factor (VEGF), but also integrates inflammatory signaling with metabolic reprogramming, thereby establishing a multilevel regulatory loop. In contrast to single-target effector molecules, therapeutic targeting of HIF-1α enables a more systematic disruption of the pathological cascade underlying RA. Collectively, these properties position HIF-1α at the center of the molecular network governing pathological angiogenesis in RA, making it a highly attractive therapeutic target (Yfantis et al., 2023). By activating mediators of pathological angiogenesis, HIF-1α accelerates the disease progression of RA (Guo and Chen, 2020). In recent years, regulating angiogenesis with targeting HIF-1α has emerged as a novel therapeutic strategy for RA. Among these, natural botanical drugs containing multi-component, multi-target metabolites from Chinese medicine (CM) formulations and plant sources have demonstrated unique potential in modulating HIF-1α-mediated angiogenesis due to their synergistic effects (Sparks, 2019). This article aims to elucidate the molecular regulatory mechanisms of HIF-1α-mediated angiogenesis and the research progress in natural botanical drugs interventions, providing directions for future research strategies (Figure 1).

## 2 Methods

### 2.1 Literature search strategy

To summarize the current state of research on the use of natural medicines to modulate HIF-1α-mediated angiogenesis pathways as an intervention for RA, this study conducted a literature search using the PubMed (https://pubmed.ncbi.nlm.nih.gov/) and CNKI (https://www.cnki.net/) databases. The search strategy used keywords such as “traditional Chinese medicine,” “natural medicines,” “rheumatoid arthritis,” “RA,” “HIF-1α,”“angiogenesis,” among others, with the following search query: (“rheumatoid arthritis“ OR “RA”) AND (“HIF-1α” OR “hypoxia-inducible factor”) AND (“angiogenesis“ OR “vascularization”) AND (“traditional Chinese medicine” OR “natural product” OR ‘phytochemical’ OR “Chinese medicine” OR “natural product”), with a search period spanning from the database’s inception to December 2025. Literature screening followed these principles: exclusion of duplicated publications, conference abstracts, master’s and doctoral theses, and studies unrelated to HIF-1α-mediated angiogenesis or natural products; for studies on natural botanical drugal formulas, exclusion of literature where the composition of the metabolite formula could not be clearly identified, as well as studies with obvious flaws in experimental design. The final inclusion criteria required original studies with rigorous experimental designs; for studies on natural botanical drugal formulas, the identification of active components was mandatory. Additionally, both the botanical drugal formulas and their individual components had to exert their therapeutic effects on rheumatoid arthritis by modulating the HIF-1α-mediated angiogenesis pathway. This study is not a systematic review; therefore, it did not follow the PRISMA guidelines to create a flowchart, nor did it conduct a methodological quality assessment of the included studies or a quantitative meta-analysis.

### 2.2 Comprehensive method for assessing the quality of literature evidence

Pan-assay interference compounds (PAINS) represent a pervasive challenge in the screening of bioactive compounds, particularly within natural products research, posing a serious threat to the reliability of research conclusions (Magalhães et al., 2021). In view of this, the present study conducted PAINS screening on all bioactive compounds included in the analysis (see Table 1 for details). However, PAINS screening alone is insufficient for a comprehensive evaluation of study quality, as it lacks the capacity to assess the risk of internal bias across studies and the hierarchical differences among evidence sources.

**TABLE 1:** PAINS Risk assessment criteria for active metabolites of natural medicines.

Risk level	Judgment criteria	Treatment measures
High Risk	Possess definite PAINS substructures and are known non-natural products	Exclude actively and exclude from evidence grade evaluation
Medium Risk	Exhibit potential PAINS-like reactive characteristics	Downgrade the evidence level by one grade (e.g., A+ → A^−^)
Low Risk	Free of known PAINS substructures, or with direct target binding verification	Retain and evaluate according to the original evidence grade

To address this shortcoming, this study introduced an adapted version of the Grading of Recommendations Assessment, Development, and Evaluation (GRADE) framework. The original GRADE system is primarily applicable to systematic reviews of clinical intervention studies (Langer et al., 2012). However, since research into the mechanisms of natural products is primarily driven by the validation of molecular targets, and the evidence is derived from multiple levels—including clinical samples, animal and cell experiments, database screening, and literature reviews—with most studies lacking a unified clinical endpoint, the original GRADE framework is difficult to apply directly. Therefore, this study adapted the GRADE framework to better suit the specific characteristics of natural product research (see Table 2). Specifically, the traditional “study design” hierarchy was replaced by a “source of mechanism” hierarchy, establishing a four-tier evidence system ranging from clinical samples (Grade A) to literature reviews (Grade D). Evidence reliability was further stratified based on the “comprehensiveness of mechanism validation,” resulting in an eight-tier classification from A^+^/A^−^ to D^+^/D^−^. In addition, a mechanism for downgrading or upgrading evidence was developed based on study-specific conditions: ① Studies in which the mechanism originates from computational biology/bioinformatics analysis but subsequent validation uses clinical samples for mechanism verification are classified as Grade A+. ② Studies that employ two or more methods followed by experimental validation are upgraded by one level. ③ Studies that combine component identification (e.g., based on mass spectrometry) with computational biology/bioinformatics analysis are upgraded by one level. ④ Studies that perform only mechanism prediction without subsequent experimental research are directly classified as Grade D−. It should also be noted that the modified GRADE framework used in this review is intended solely for the retrospective assessment of the quality and reliability of included studies, aiming to evaluate their methodological rigor. This assessment does not influence the literature screening process and is not used as a criterion for the inclusion or exclusion of studies.

**TABLE 2:** Grading evaluation criteria for mechanism studies of diseases intervened by natural medicines.

Evidence grade	Mechanism source	Verification completeness	Evaluation standard
A^+^	Clinical samples	Complete	Derived from clinical samples, verified by both *in vivo* and *in vitro* experiments
A^-^	Clinical samples	Partial	Derived from clinical samples, verified by either *in vivo* or *in vitro* experiment alone
B^+^	Animals/Cells	Complete	Derived from animal or cellular experiments, verified by both *in vivo* and *in vitro* experiments
B^−^	Animals/Cells	Partial	Derived from animal or cellular experiments, verified by either *in vivo* or *in vitro* experiment alone
C^+^	Computational Biology/Bioinformatics	Complete	Screened from databases, verified by both *in vivo* and *in vitro* experiments
C^−^	Computational Biology/Bioinformatics	Partial	Screened from databases, verified by either *in vivo* or *in vitro* experiment alone
D^+^	Literature	Complete	Summarized from published literature, verified by both *in vivo* and *in vitro* experiments
D^-^	Literature	Partial	Summarized from published literature, verified by either *in vivo* or *in vitro* experiment alone

Based on the aforementioned modified framework, this study first subjected the single active ingredients from the included natural products to PAINS screening to determine whether they contained any known high- or medium-risk PAINS compounds. Subsequently, the modified GRADE framework was applied to grade the quality of evidence. For complex herbal formulae containing multiple active ingredients, this study did not exclude them outright merely because they contained PAINS compounds; however, PAINS screening was still conducted on their constituent components, applying the following specific handling principles:① If no PAINS compounds were detected in the formulae, or if the PAINS compounds were not the primary active ingredients responsible for the therapeutic effect, the formulae were included as normal and their original evidence grade was retained.② If the primary active ingredient of a compound was identified as a high-risk or medium-risk PAINS compound, the evidence grade for that compound was downgraded by one level from the original.③ If all reported active ingredients in a compound were PAINS compounds, the grade was downgraded by two levels or directly assigned a D^−^ grade. The aforementioned preliminary screening and grading process ensures that the evidence base upon which this review is founded possesses acceptable reliability.

## 3 The molecular network and mechanisms of HIF-1α-mediated angiogenesis in RA

### 3.1 HIF-1α and RA angiogenesis

HIF-1α plays a crucial role in regulating abnormal synovial metabolism, angiogenesis, and the proliferation and apoptosis of synovial cells (Cai et al., 2023; Ghosh et al., 2022). Under normal physiological conditions, the most fundamental response to local hypoxia is the induction of neovascularization or angiogenesis from existing blood vessels to supplement oxygen supply to ischemic or damaged tissues. Angiogenesis is primarily initiated by pro-angiogenic substances, particularly VEGF, through the activation of proliferation and migration in vascular endothelial cell (VEC). HIF-1α plays a dominant role throughout the angiogenesis process. As the primary transcriptional mediator of the hypoxia response and the main regulator of O_2_ homeostasis, HIF-1α expression increases when there exists an imbalance between O_2_ supply and demand due to the oxygen concentration decrease (Ortmann et al., 2024). During this process, significant structural changes occur in HIF-1α structural domain: with the inhibition of PHD and FIH activities and reduced affinity between HIF-1α and pVHL, HIF-1α loses hydroxylation modifications, and its hydroxylation reactions gets suppressed. Whereafter,the stability of HIF-1α expression increases, and HIF-1α is translocated into the nucleus (Bruick and McKnight, 2001)to form a dimer on the HIF response element (HRE) by binding with HIF-1β. (Zhang et al., 2014; Baek and Kim, 2016; Befani and Liakos, 2018), thereby activating downstream target gene transcription and then activating the transcritional levels of angiogenesis-promoting factors such as VEGF and Angiopoietin (ANGPT), promoting the proliferation and migration of vascular endothelial cells and inducing neovascularization (Quiñonez-Flores et al., 2016) and improving the local oxygen environment, so that cellular energy metabolism can be maintained, and cells, tissues, and species can adapt to hypoxic conditions (Sabi et al., 2022). HIF-1α serves as a critical hub connecting the cellular microenvironment with the body’s adaptive responses by integrating oxygen concentration signals and regulating VEGF-mediated angiogenesis. In RA, hypoxia leads to abnormally high expression of HIF-1α in RA synovial tissues. Under the influence of HIF-1α, FLS, as key effector cells, can increase the expression levels of pro-angiogenic factors such as VEGF and ANG, which bind to corresponding receptors on VEC, promoting VEC proliferation and directly or indirectly facilitating angiogenesis (Chen Zifeng et al., 2025).

### 3.2 Core regulatory factors and molecular networks of HIF-1α-mediated angiogenesis

In the synovial tissue of RA, the inflammation and hypoxic microenvironment significantly promotes neovascularization and enhances the release of vascular growth factors, chemokines, and factors related to the glucose metabolic pathway, thereby exacerbating disease progression (Zheng Q. et al., 2025) (Table 3, Figure 3).

**TABLE 3:** Core mediators and pathways of HIF-1α-regulated RA angiogenesis.

Path level	Factor/Pathway type	Core molecule	Primary functions in RA angiogenesis	Regulatory role of HIF-1α	References
Primary Core	GF	VEGF	Promote VEC proliferation, migration, and lumen formation; increase vascular permeability	Direct transcription activates the VEGF gene; induces macrophages/FLS to secrete VEGF	Li et al. (2018); Ba et al. (2021)
		ANG-1/2	ANG-1 maintains vascular stability; ANG-2 disrupts stability under pathological conditions, promotes vascular sprouting, and synergizes with VEGF	Transcriptionally activates the ANGPT2 gene; ANG-2 synergistically promotes angiogenesis with VEGF	Chang et al. (2021), Befani and Liakos (2018)
Secondary coordination	Chemotactic factor	CXCL12/CXCR4	Chemoattract inflammatory cells; promotes angiogenesis by directly or indirectly inducing VEGF	Hypoxia directly upregulates CXCL12 expression; the CXCL12/CXCR4 axis further amplifies VEGF signaling	Janssens et al. (2018), Da et al. (2023)
	Signal channel	PI3K/Akt/mTOR	Promote cell survival, proliferation, and protein synthesis; positive feedback enhances HIF-1α protein translation	HIF-1α is a critical upstream activation pathway; its downstream effects are partially mediated by HIF-1α	Lyons and Roche (2018), Zou et al. (2016)
Three stage amplification	Developmental signal	Notch1/3	Notch1 regulates FLS migration/EMT; Notch3 regulates anti-apoptosis/autophagy; both synergistically promote hypoxia-induced invasion and angiogenesis	Bidirectional regulation: (Díaz-González and Hernández-Hernández, 2023) HIF-1α transcriptionally activates Notch1/3; (Radu and Bungau, 2021) Notch signaling enhances the stability of HIF-1α protein	Zhuang et al. (2022), Wei et al. (2020)
	Inflammatory signals	TLR4/HMGB1	As a DAMP, HMGB1 activates inflammation via TLR4, upregulates HIF-1α expression, and promotes VEGF secretion	HMGB1-TLR4 forms a positive feedback loop with HIF-1α	Konisti et al. (2012), Park et al. (2015)
	Metabolic reprogramming	Glycolysis/Lactic Acid	It provides energy for the proliferation of FLS, VEC, and immune cells; lactic acid accumulation stabilizes HIF-1α, forming a metabolic-hypoxic positive feedback loop	Transcriptionally upregulates genes of glycolytic enzymes such as GLUT1, HK2, PFKFB3, LDHA, and PDK1	Wei and Yu (2007), Shen et al. (2012)

#### 3.2.1 HIF-1α/VEGF/ANG

HIF-1α-mediated synovial neovascularization depends on multiple mediators, among which VEGF and ANG are considered as the primary mediators and key signaling pathways in angiogenesis. VEGF is a cytokine that acts on synovial vascular endothelial cells. By binding to its specific receptors, it activates vascular endothelial cells, promotes angiogenesis, and enhances the production of proteolytic enzymes (Melincovici et al., 2018). It serves as the primary target of HIF-1α in regulating angiogenesis, with its expression being hypoxia-dependent. The ANG family is a crucial group of proteins that regulate angiogenesis and vascular stability. Among them, ANG-1 and ANG-2 can modulate the stability of VECs (Pei-rong et al., 2021). They play a pivotal role in synovial angiogenesis and the maintenance of vascular stability in RA and are also important regulatory factors in HIF-1α-mediated angiogenesis. HIF-1α not only stimulates synovial macrophages and fibroblasts to secrete VEGF but also upregulates VEGF by promoting the proliferation of epithelial-mesenchymal transition factors, collectively driving the formation of new blood vessels in the synovium (Ao et al., 2022). In RA synovial tissues, elevated HIF-1α expression typically drives the transcriptional activation of VEGF, increasing synovial vascular permeability and promoting synovial angiogenesis (Li et al., 2018). Studies have shown (Ba et al., 2021) that VEGF reduces VEC apoptosis while promoting their proliferation, migration, and lumen formation. Simultaneously, VEGF induces inflammatory responses, where synovial inflammation interacts with angiogenesis, exacerbating inflammatory cell migration and panniculosis formation, ultimately damaging adjacent cartilage and bone. Ang-1 maintains the stability of neovascular VEC and restricts abnormal vascular formation. Research has found (Chang et al., 2021) that Ang-1 is highly expressed in synovial fluid of RA patients, Futher *in-vivo* experiments have also shown that inhibiting its expression reduces angiogenesis and joint swelling in the ankle joint of collagen-induced arthritis (CIA) animal model. In contrast, Ang-2 promotes abnormal vascular formation and participates in vascular remodeling and degeneration. It is closely associated with VEGF and exhibites a pronounced VEGF-dependent mechanism. In RA, Ang-2 upregulates angiogenic markers such as CD31 and CD34 by competitively binding to Tie-2, synergizing with VEGF to promote angiogenesis (Befani and Liakos, 2018). HIF-1α regulates the synergistic angiogenic effects of VEGF and ANG. HIF-1α accelerates angiogenesis by upregulating VEGF expression, while regulating ANG to promote vascular maturation and maintain angiogenic stability.

#### 3.2.2 HIF-1α/CXCL12/CXCR4

CXC chemokines exhibit pleiotropic effects in immune regulation and angiogenesis (Strieter et al., 2005). Matrix cell-derived chemokine-1α (CXCL12), a member of the CXC chemokine family, is derived from immune cells and endothelial cells, playing a critical role in maintaining homeostasis of the internal environment (Wu et al., 2021). CXCL12 is expressed in hypoxic and angiogenic environments of autoimmune diseases, and its expression is enhanced with the upregulation of HIF-1α. CXCL12 exerts its effects by activating its receptor, CXC chemokine receptor 4 (CXCR4) (Janssens et al., 2018). The interaction between CXCL12 and endothelial CXCR4 also promotes the production of VEGF. Studies have shown that in the synovial tissues of RA, the expression of HIF-1α is significantly increased under hypoxic conditions, which can regulate the CXCL12/CXCR4 axis to enhance VEGF production, thereby contributing to angiogenesis and panniculosis formation in RA synovium.

#### 3.2.3 HIF-1α/glycemic metabolic pathway

The “inflammation-hypoxia” microenvironment is the predominant state of synovial tissue in RA. This “inflammation-hypoxia” microenvironment drives metabolic reprogramming in RA FLS, shifting from oxidative phosphorylation to aerobic glycolysis (Warburg effect, a process regulated by HIF-1α as the core transcription factor (Zheng L,AX. et al., 2025; Jia et al., 2025; Aghakhani et al., 2022; Wu et al., 2026). Glycolysis-generated adenosine triphosphate (ATP) provides energy for local immune and interstitial cell proliferation and activation, while VEC rely on glycolytic energy production to maintain vascular homeostasis. High glycolytic activity leads to significant lactate accumulation, which inhibits PHD activity, thereby preventing HIF-1α hydroxylation and degradation, thus stabilizing HIF-1α protein (Wu et al., 2026). This “glycolysis-lactate-HIF-1α” positive feedback loop maintains high HIF-1α expression in synovial tissue even under normoxic conditions, continuously driving angiogenesis and inflammation (Lyons and Roche, 2018). HIF-1α enhances glycolysis dependence of VEC under hypoxic conditions and promotes VEC angiogenesis by mediating the expression of proteins involved in cellular hypoxia responses (Wei and Yu, 2007). Inhibiting glycolysis can disrupt the HIF-1α-driven pathological cycle. 3-Bromopropionic acid (3-BrPA) or HK2 knockdown significantly suppresses macrophage interleukin-1 beta (IL-1β) production and reduces HIF-1α levels. Studies have shown (Shen et al., 2012) that restoring pyruvate synthesis capacity by inhibiting lactate dehydrogenase (LDH) significantly decreases HIF-1α and VEGF expression in the joints of adjuvant-induced arthritis (AIA) rats, alleviates inflammation-related hypoxia, and impedes pathological neovascularization. Therefore, targeting HIF-1α-mediated glycolytic reprogramming becomes a key strategy in RA anti-angiogenic therapy.

#### 3.2.4 PI3K/akt/HIF-1α

In RA, the PI3K/Akt signaling pathway plays a crucial role in HIF-1α-mediated angiogenesis. The stability of HIF-1α is associated with the PI3K/Akt signaling pathway under hypoxic conditions (Xie et al., 2019). The protein levels of HIF-1α are regulated by the PI3K/Akt pathway, and the phosphorylation levels of PI3K and Akt are inhibited, leading to downregulation of HIF-1α expression (Xiao et al., 2023). PI3K inhibitors could reduce HIF-1α expression. Studies have shown (Zou et al., 2016) that HIF-1α activates the PI3K/Akt signaling pathway and promotes neovascularization. Hypoxia regulates VEGF induction by activating the stress-induced PI3K/Akt pathway and HIF-1α. Additionally, HIF-1α is influenced by mTORC1, a downstream target of the PI3K/Akt pathway. PI3K can activate mTOR through AKT phosphorylation, thereby upregulating HIF-1α expression. Research has revealed that interrupting the PI3K/AKT/mTOR/HIF-1 pathway can inhibit angiogenesis in RA (Lyons and Roche, 2018).

#### 3.2.5 HIF-1α/notch

The HIF-Notch signaling pathway has been demonstrated to mediate angiogenesis in various diseases (Yang et al., 2017). Notch1/3 in the Notch signaling pathway are significantly expressed in RA synovial tissues and play a critical role in hypoxia-induced synovial lesions. Functionally, Notch1 primarily regulates cell migration and epithelial-mesenchymal transition, enhancing the invasive capacity of FLS and synovial hyperplasia. Notch3 mainly regulates cell survival, anti-apoptosis, and autophagy, maintaining FLS viability in the hypoxic microenvironment. Both of them synergistically drive hypoxia-induced RA FLS invasion and pathological angiogenesis (Zhuang et al., 2022). Studies have clearly revealed the mutual activation relationship between HIF-1α and the Notch signaling pathway, forming a bidirectional positive feedback regulatory loop: under hypoxic conditions, HIF-1α directly regulates the expression of Notch-1 and Notch-3 genes (Chen et al., 2021a). The Notch signaling pathway inhibits the hydroxylation of HIF-1α and its recruitment to specific sites, thereby enhancing the stability of HIF-1α and its protein stability, forming a vicious cycle of “hypoxia-HIF-1α→Notch1/3→HIF-1α stability” (Wei et al., 2020; Kim et al., 2020). Notch1/3 activation, through forming a bidirectional positive feedback loop with HIF-1α, collaboratively upregulates pro-angiogenic factors such as VEGF and ANG, and enhances the migratory, invasive, and survival capabilities of FLS, driving pathological angiogenesis and synovial hyperplasia in RA. Previous studies have demonstrated that HIF-1α siRNA can inhibit the expression of Notch-1 intracellular domain (N1ICD) and Notch-3 intracellular domain (N3ICD) in RA FLS exposed to hypoxia (Chen et al., 2021b). Research has also shown (Šućur et al., 2020) that γ-secretase inhibitors can suppress the proliferation of RA FLS, the secretion of inflammatory cytokines, and angiogenesis, while blocking Notch1 and Notch3 signaling, which significantly alleviates arthritis symptoms and disease severity in collagen-induced arthritis (CIA) rats.

#### 3.2.6 TLR/HMGB1/HIF-1α

Toll-like receptors (TLRs) drive the inflammatory cytokines tumor necrosis factor-alpha (TNF-α), interleukin-6(IL-6), and interleukin-18(IL-18), which are secreted by M1 macrophages, to combine with the increased intracellular expression of HIF-1α activated under local hypoxic conditions, activating RA macrophages and synovial tissue fibroblasts to secrete growth factors and promoting synovial angiogenesis (Konisti et al., 2012). There exists a bidirectional regulatory relationship between HIF-1α and TLR4 in the Toll-like receptor family. On one hand, TLR4 activation induces HIF-1α expression through the nuclear Factor-kappa B(NF-κB) pathway; on the other hand, HIF-1α can directly transcribe to upregulate TLR4 expression, forming a positive feedback amplification loop. The pro-inflammatory state induced by autoantibodies in RA, along with elevated levels of cytokines and chemokines, sensitizes sensory neurons, thereby triggering pain responses. This demonstrates TLRs are closely associated with chronic pain (Kato et al., 2016). Stimulation by TLR ligands sensitizes nociceptors to other pain stimuli. Toll-like receptor 4 (TLR4) is expressed on nociceptors, and the HIF-1α-related pathway can upregulate TLR4 expression in macrophages (Kim et al., 2010). The neovascularization of synovial endothelial fibroblasts in RA is accompanied by the neurogenesis of nociceptive axons, collectively enhancing inflammatory and nociceptive signaling (Sow and Hasegawa, 2025). Therefore, the HIF-1α/TLR4 positive feedback loop serves as a key mechanism linking the hypoxic microenvironment to innate immune responses, providing dual therapeutic targets for RA treatment.

As a ligand of TLR4, high-mobility group box chromosomal protein 1 (HMGB1) is a non-histone nuclear protein and cytokine mediator. And is abundantly extracellularly present in synovitis. Hypoxia increases extracellular HMGB1 expression, serving as a critical coupling factor between hypoxia and inflammation. Patients with RA exhibit higher extracellular HMGB1 expression in synovial tissue than those with osteoarthritis. Under hypoxic conditions, HMGB1 enhances the expression level and activity of HIF-1α in FLS from RA patients, promoting VEGF expression. HMGB1-mediated VEGF production is dependent on HIF-1, and HMGB1 stimulates the HIF-1α/VEGF axis to promote angiogenesis (Hong et al., 2022). Treatment of RA synovial fibroblasts with HMGB1 elevates HIF-1α expression and HIF-1 activity (Park et al., 2013). Studies have shown (Park et al., 2015) that inhibiting HMGB1 may demonstrate potential benefits in treating RA-related angiogenesis, revealing HMGB1’s involvement in HIF-1-mediated angiogenesis processes.

HIF-1α interacts through multiple pathways and collaboratively regulates angiogenic activities within vascular cells, playing a pivotal role in synovial angiogenesis during RA. As the “master switch” sensing hypoxia, HIF-1α not only directly upregulates the transcription of pro-angiogenic factors such as VEGF and ANG but also directly activates VEGF, initiating the proliferation and migration of vascular endothelial cells. This establishes the HIF-1α/VEGF/ANG signaling axis as the core driver of synovial angiogenesis in RA. Additionally, HIF-1α provides energy support for the activation and proliferation of FLS, VEC, and immune cells through modulating glycolysis to reshape cellular metabolism, (Fearon et al., 2022). It also indirectly amplifies angiogenic effects by interacting with signaling molecules such as chemokines and HMGB1, etc. The PI3K/Akt/mTOR pathway enhances the protein synthesis of HIF-1α to amplify its effects. The Notch signaling pathway regulates the differentiation of tip cells in vascular sprouting to determine the morphology of neovascularization. The CXCL12/CXCR4 axis promotes angiogenesis by chemotactically recruiting inflammatory cells and inducing VEGF production. TLR couples inflammation with neuroimmunity, while HMGB1 links inflammatory signals with hypoxic responses, forming a positive feedback regulation. The above hierarchical regulatory network collectively constitutes a complex system centered on HIF-1α, driving the formation and progression of synovial pannus in RA. These core pathways and mediators collectively form a sophisticated regulatory network centered on HIF-1α, continuously driving the development of pannus and joint structural damage. These findings provide therapeutic targets and directions for regulating HIF-1α signaling to inhibit synovial angiogenesis and alleviate RA symptoms.

### 3.3 Mechanisms of HIF-1α-mediated angiogenesis in RA

**FIGURE 2:**
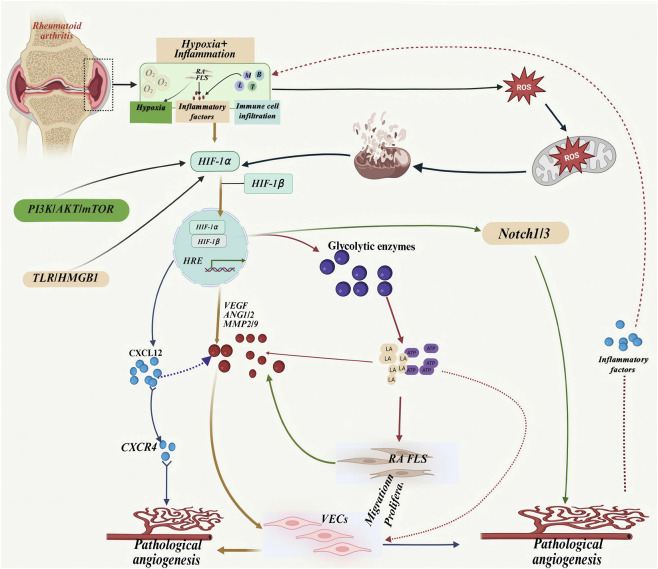
Diagram of the core regulators of HIF-1α driving RA angiogenesis and their molecular network with the core hub mechanism (Díaz-González and Hernández-Hernández, 2023). Immune cell infiltration, hypoxia, and the inflammatory microenvironment in the RA synovium lead to the stabilization of HIF-1α protein. Subsequently, HIF-1α translocates to the nucleus to form a transcriptionally active dimer with HIF-1β. Activated HIF-1α initiates the transcription of multiple downstream signals by binding to HRE: ① Upregulates the expression of VEGF and ANG-1/2, which are the primary direct drivers of VEC proliferation, migration, and lumen formation; ② Upregulates CXCL12, which promotes VEGF expression or angiogenesis through the CXCR4 receptor; ③ Enhances the glycolytic pathway, providing essential energyof ATP for angiogenesis and related cellular activities. Activation of the glycolytic pathway promotes the proliferation and migration of VEC and FLS, and increases the expression of angiogenic factors, further promoting neovascularization (Radu and Bungau, 2021). HIF-1α is also regulated by upstream PI3K/Akt/mTOR and TLR/HMGB1 pathways, which are activated by microenvironmental factors and provide positive feedback to enhance the stabilization and expression of HIF-1α, forming an amplified signaling network (Elshabrawy et al., 2015). Immune cell infiltration, hypoxia, and the inflammatory microenvironment in the synovial membrane lead to a large accumulation of ROS resulting in mitochondrial damage; damaged mitochondria, in turn, produce more ROS, creating a vicious cycle. ROS are not only damaging agents but also important signaling molecules that stabilize and activate HIF-1α. Activated HIF-1α can directly upregulate VEGF and also regulate the Notch signaling pathway, promoting angiogenesis through this combined mechanism (Maeda et al., 2022). Neovascularization continuously delivers inflammatory cytokines to new blood vessels, further amplifying the inflammatory response, exacerbating hypoxia, promoting immune cell activation and infiltration, and further increasing the abnormally high expression of HIF-1α. HIF-1α is central to the core network of “hypoxia-metabolism-oxidation,” integrating signals from hypoxia, inflammation, and oxidative stress. It promotes pathological angiogenesis through synergistic effects by driving the inflammatory cycle, reprogramming cellular metabolism, and responding to oxidative stress (Created with BioRender (https://app.biorender.com/)).

The proliferation of the synovial endomembrane layer and the recruitment of immune cells lead to synovial inflammation and hypoxic conditions (Taylor and Sivakumar, 2005). In the angiogenesis process of RA, HIF-1α plays a critical regulatory role (Acuña-Pilarte and Koh, 2025). HIF-1α-mediated angiogenesis drives the formation of a “hypoxia-metabolism-oxidation” microenvironment in rheumatoid arthritis by regulating pathological processes including synovial inflammatory responses, glycolytic metabolic reprogramming, and oxidative stress-induced mitochondrial damage, thereby promoting the further development of synovial pannus (Quiñonez-Flores et al., 2016; Wang et al., 2021) (Figure 2).

#### 3.3.1 Hypoxia-driven inflammatory-vascular vicious cycle

The inflammatory and hypoxic microenvironment constitutes the primary pathological state of RA synovium (Muz et al., 2009). In RA, angiogenesis is predominantly mediated by the interaction of fibroblast-like cells (FLS), VEC, and macrophages within synovial tissue (Zhang et al., 2021). Neovascularization is typically associated with hypoxia in the pathological synovial state of arthritis (Zaripova et al., 2021; Giatromanolaki et al., 2003). In RA, hypoxia induces abnormally high expression of HIF-1α in synovial tissues,and FLS consequently undergoes abnormal activation and transforms into RA FLS with an aggressive phenotype that promotes arthritis progression (Ma et al., 2025). HIF-1α further induces the expression of VEGF, ANG, matrix metalloproteinases (MMPs), and stromal cell-derived factor-1 (SDF-1), thereby indirectly promoting angiogenesis (Konisti et al., 2012). The newly formed vasculature continuously delivers large numbers of inflammatory cells such as T cells, B cells, and macrophages, along with pro-inflammatory factors like TNF-α, IL-1β, and IL-6, further amplifying the inflammatory response, and then maintaining the inflammatory microenvironment (Leblond et al., 2017), exacerbating inflammatory cell infiltration in synovial tissues and aggravating cartilage and bone injury. Concurrently, synovial inflammation leads to excessive proliferation and hypertrophy of synovial tissue. The proliferation of RA FLS and active immune cells in synovial tissues increases the demand for oxygen and nutrients. However, the formation of new blood vessels often fails to meet the oxygen requirements of synovial tissues, resulting in reduced local microenvironmental oxygen tension and the establishment of a hypoxic synovial microenvironment (Chen S. et al., 2023). The exacerbation of hypoxic conditions leads to persistent and significantly elevated abnormal expression of HIF-1α. So repeatedly, there forms a vicious cycle like “hypoxia-FLS/VEC proliferation-angiogenesis-hypoxia…” (Zimna and Kurpisz, 2015). Additionally, inflammatory cytokines can directly stabilize HIF-1α protein through pathways such as NF-κB and PI3K/Akt, thereby promoting angiogenesis even under normoxic conditions (Konisti et al., 2012). The interaction between angiogenesis and synovial inflammatory responses jointly promotes the formation of pannus, thereby mediating the progressive destruction and functional impairment of joint structures.

#### 3.3.2 Metabolic reprogramming

HIF-1α is a key regulatory factor in cellular metabolic reprogramming. The metabolic reprogramming mediated by HIF-1α, such as glycolysis, provides energy for the proliferation and migration of FLS and VEC, further inducing angiogenesis (Li et al., 2018). Under the pathological state of RA, the expression of HIF-1α is upregulated, leading to an increased expression of various glycolytic enzymes. This results in a metabolic functional shift in FLS and VEC, enabling them to generate sufficient energy to support synovial proliferation and angiogenesis (Kierans and Taylor, 2021; Peng et al., 2021). The hypoxic microenvironment of RA synovium enhances glycolytic activity. This hypoxic microenvironment stabilizes HIF-1α and transcribes and activates key genes of glycolytic enzymes, driving the “Warburg effect,” so that cells can rapidly generate ATP through glycolysis and provide precursors for biosynthesis to meet the energy demands of FLS and VEC for proliferation and migration (Li et al., 2018). Meanwhile, lactate produced by glycolysis prevents the degradation of HIF-1α through inhibiting PHD activity, thereby forming a self-reinforcing positive feedback loop of “glycolysis-lactate-HIF-1α.” This loop allows RA synovium to maintain high HIF-1α expression even under normoxic conditions, continuously driving the release of VEGF and Matrix metalloproteinase-2/9 (MMP-2/9). Furthermore, glucose-6-phosphate isomerase (GPI) is a key enzyme involved in the “Warburg effect” of RA glycolysis and is closely related to RA activity (Xu et al., 2020). Hypoxic conditions can upregulate GPI activity. In RA synovial tissue cells, the upregulated GPI can induce RA angiogenesis by increasing the expression of HIF-1α and VEGF (Lu et al., 2017).

#### 3.3.3 Oxidative stress-mitochondrial damage axis

In the hypoxic and inflammatory microenvironment of RA, hypoxia and inflammation in synovial tissue lead to the accumulation of reactive oxygen species (ROS). ROSs damage mitochondria and promote oxidative stress responses. Mitochondrial injury and oxidative stress in synovial tissue are key factors inducing angiogenesis (Lóp et al., 2022; West, 2017). HIF is the primary regulator responding to ROS and mediating angiogenesis. ROS accumulation in RA joints not only promotes the high expression of HIF, including HIF-1α and HIF-2α, but also further stabilizes HIF-1α by upregulating VEGF expression through HRE (Willson et al., 2022). These free radicals are subsequently converted into hydrogen peroxide via superoxide dismutase (SOD) and further promote the formation of lipid peroxidation products, thereby enhancing VEGF expression and driving angiogenesis in RA. Additionally, the Notch signaling pathway can regulate and control ROS levels. The Notch pathway, regulated by HIF can inhibit Notch-induced proliferation, migration, and adhesion of human umbilical vein endothelial cells (HUVEC), by clearing ROS in HUVEC. Furthermore, mutations in mitochondrial ubiquinol-cytochrome C reductase-binding protein (UQCRB), one of the subunits of mitochondrial respiratory chain complex III, increase mtROS production and activate HIF-1α trans-activation, promoting angiogenesis (Reichard and Asosingh, 2019; Chang et al., 2014).

## 4 Natural product interventions mediating HIF-1α-induced RA angiogenesis

In recent years, natural botanical drugs, including CM metabolite formulations and plant-derived monomeric metabolites, have demonstrated unique potential in the treatment of RA. Their core advantages lie in multi-target and holistic regulatory characteristics, enabling simultaneous intervention at multiple links within the HIF-1α signaling network, thereby more effectively breaking the vicious cycle of “hypoxia-inflammation-angiogenesis.” These metabolites show broad prospects in intervening HIF-1α inhibition to reduce angiogenesis in RA and alleviate disease progression. CM metabolite formulations achieve comprehensive intervention in the pathological process of RA through synergistic effects of multiple components, regulating autophagy-apoptosis balance, “synovial cell-endothelial cell” crosstalk, and multiple signaling pathways. Monomeric metabolites exert anti-inflammatory and anti-angiogenic effects by precisely targeting the HIF-1α-related axis, cellular metabolism, or signaling pathways. These studies provide molecular-level evidence for CM treatment of RA and highlight the importance of HIF-1α-mediated angiogenesis as a therapeutic target for RA (Tables 4, 5).

**TABLE 4:** Preclinical studies on Chinese medicine compound formulas targeting HIF-1α-mediated angiogenesis in the treatment of rheumatoid arthritis.

Compound name	Composition		Active ingredients	Model/Cell	Induction method	Range of dosage	Key effects	Molecular mechanism	Evidence level	References
	Original literature	POWO and MPNS verification								
Fangji Huangqi Decoction	Stephania tetrandra, Astragalus membranaceus, Astragalus membranaceus; Glycyrrhiza uralensis, Zingiber, Ziziphus jujuba	Botryodiscia tetrandra (S. Moore) L. Lian & Wei Wang, Astragalus mongholicus Bunge, Glycyrrhiza uralensis Fisch. ex DC., Zingiber zerumbet (L.) Roscoe ex Sm., Ziziphus jujuba subsp. Spinosa (Bunge) J. Y. Peng, X. Y. Li and L. Li	Fangchinoline, Liquiritigenin, Phellodendrine, L-Hyoscyamin, and Liquiritin	*In vivo*: Male SD rats with CIA *In vitro*: RA-FLS, HUVEC	Primary immunization: BCII combined with CFA Secondary immunization: BCII combined with CFA on the 7th day after primary immunization	*In vivo*: 4.4 g/kg/day for 4 weeks *In vitro*: Rats were intragastrically administered with FHD at 22 g/kg for 7 days, blood was collected 2 h after the last administration to separate serum, and 2.5%, 5% and 7.5% drug-containing serum were used	① ↓ Arthritis index and foot swelling; ② ↓ Synovial hyperplasia, cartilage damage and vascular density; ③ ↓ MH7A proliferation, migration and invasion; ④ ↓ MH7A-induced HUVEC tubule formation; ⑤ ↓ CD31, VEGF and vimentin expression; ⑥ ↓ HIF-1α expression; ⑦ ↓ LC3B-II/I ratio, ↑ p62; ⑧ ↑ Bax, Caspase-3, ↓ Bcl-2	Inhibit HIF-1α to regulate the balance between autophagy and apoptosis, and restrain FLS-induced angiogenesis	C+	Peng et al. (2021)
Huayu Tongbi formula	Radix Salviae, Rhizoma Dioscoreae Nipponicae, Hedysarum Multijugum Maxim, Aconiti Lateralis Radix Praeparata, Paeoniae Radix Alba, Corydalis Rhizoma, licorice	Salvia miltiorrhiza Bunge, Dioscorea nipponica Makino, Corethrodendron multijugum (Maxim.) B. H. Choi and H. Ohashi, Aconitum carmichaelii Debeaux, Paeonia lactiflora Pall., Corydalis yanhusuo (Y. H. Chou & Chun C. Hsu) W. T. Wang ex Z. Y. Su and C. Y. Wu, Glycyrrhiza uralensis Fisch. ex DC.	N-acetylornithine, sulfamethazine, quinic acid, phenylacetaldehyde, 6-pentyl-2H-pyran-2-one, uridine, monobenzyl phthalate, and 3,3′,5-triiodo-l-thyronine sulfamethazine, 3,3′,5-triiodo-l-thyronine, 6-pentyl-2H-pyran-2-one, N-acetylornithine, 4 guanidinobutyric acid, l-tyrosinemethylester, 18-β-glycyrrhetinic acid, uridine, monobenzyl phthalate, indole-3-lactic acid, N-acetyl-l-histidine, quinic acid, benzaldehyde 1-(2,4-dinitrophenyl)hydrazone, phenylacetaldehyde, and aflatoxin G2	*In vivo*: Female SD rats with CIA; *In vitro*: RA-FLS, HUVEC	Primary immunization: Subcutaneous injection of 0.2 mL BCII combined with IFA at the tail root; Secondary immunization: Subcutaneous injection of 0.1 mL BCII combined with IFA at the tail root on the 7th day after primary immunization	*In vivo*: 6.35, 12.71, 25.41 g/kg/d for 4 weeks; *In vitro*: Intragastric administration of HT at 38.12 g/kg/d twice a day for 7 days, blood was collected 1 h after the last administration, and 5%, 10% and 15% drug-containing serum were used for cell treatment	① ↓ Arthritis index and plantar thickness; ② ↓ Bone destruction and pathological scores; ③ ↓ NF-α and L-1β expression; ④ ↓ Synovial vascular density and immature vessels (CD31+/αSMA−); ⑤ ↓ Proliferation, migration, adhesion and tube formation in RA-FLS and HUVEC; ⑥ ↓ HIF1A/VEGFA/VEGFR2 protein and mRNA expression	Block the HIF1A/VEGFA/ANGPT axis, weaken the crosstalk between RA-FLS and HUVEC, thereby suppressing angiogenesis	C+	Lu et al. (2017)
Wutou Decoction	Aconitum carmichaeli Debeaux., Ephedra sinica Stapf, Paeonia lactiflora Pall., Astragalus mongholicus Bunge, Glycyrrhiza uralensis Fisch	Aconitum carmichaelii Debeaux, Ephedra sinica Stapf, Paeonia lactiflora Pall., Astragalus mongholicus Bunge, Glycyrrhiza uralensis Fisch. ex DC.	ephedrine hydrochloride pseudoephedrine hydrochloride, paeoniflorin, verbasil-7-O-glucoside, glycyrrhizinate, glycyrrhizin	*In vivo*: Male Wistar rats with CIA; *In vitro*: MH7A cells, HUVEC	Primary immunization: Subcutaneous injection of 0.3 mL BCII combined with CFA at tail root and back; Secondary immunization: Subcutaneous injection of 0.3 mL BCII combined with CFA at tail root and back on the 7th day after primary immunization	*In vivo*: Intragastric administration at 3.75 g/kg and 7.5 g/kg once a day for 28 days; *In vitro*: Prepared into freeze-dried powder at concentrations of 1 mg/mL and 10 mg/mL	① ↓ Arthritis index and swelling; ② ↓ Pathological scores for synovial hyperplasia, vascularisation, etc.; ③ ↓ Proliferation, migration and invasion of MH7A cells; ↑ Apoptosis; ④ ↓ MH7A-induced HUVEC tubule formation; ⑤ ↓ CD31/vWF expression; ⑥ ↓VEGF/ANG1 expression in MH7A cells; ⑦ ↓VEGFR2/TEK expression in HUVECs; ⑧ ↓PI3K/p-AKT/p-mTOR/HIF-1α protein levels	Inhibit the PI3K/AKT/mTOR/HIF-1α signaling pathway, reduce the secretion of VEGF and ANG1 by MH7A cells, block the activation of VEGFR2 and TEK in HUVEC, and further restrain angiogenesis	C+	Ao et al. (2022)
ShexiangZhuifeng analgesic plaster	Artificial Musk, Aconitum kusnezoffii Reichb., Aconitum carmichaeli Debeaux, Boswellia carterii Birdw., Commiphora myrrha (T. Nees) Engl., Strychnos nux-vomica L., Eugenia caryophyllata Thunb., Cinnamomum cassia (L.) J. Presl, Schizonepeta tenuifolia (Benth.) Briq., Saposhnikovia divaricate (Trucz.) Schischk., Geranium wilfordii Maxim., Periploca sepium Bunge, *Centella asiatica* (L.) Urban, Drynaria fortunei (Kunze ex Mett.) J. Sm., Angelica dahurica (Hoffm.) Benth. and Hook. f. Ex Franch. and Sav., Kaempferia galanga L., Zingiber officinale Roscoe	Artificial Musk, Aconitum kusnezoffii Rchb., Aconitum carmichaelii Debeaux, Boswellia sacra Flück., Commiphora myrrha (T. Nees) Engl., Strychnos nux-vomica L., Syzygium aromaticum (L.) Merr. and L. M. Perry, Neolitsea cassia (L.) Kosterm., Nepeta tenuifolia Benth., Saposhnikovia divaricata (Turcz. Ex Ledeb.), Geranium wilfordii Maxim., Periploca sepium Bunge, *Centella asiatica* (L.) Urb., Lepisorus fortuni (T. Moore) C. M. Kuo Angelica dahurica (Hoffm.) Benth. and Hook. f. Ex Franch. and Sav., Kaempferia galanga L., Zingiber officinale Roscoe	Loganic acid, Racanisodamine, Neoline, Atropine, Prim-O-glucosylcimifugin, 5-O-Methylvisammioside, Benzoylmesaconine, Vanillin, Scopoletin, Xanthotoxin, Byakangelicol, Oxypeucedanin	*In vivo*: Male Wistar rats with CIA; *In vitro*: RAW264.7 cells	Primary immunization: Subcutaneous injection of 0.25 mL BCII combined with IFA at the tail root; Secondary immunization: Subcutaneous injection of 0.15 mL BCII combined with CFA at the tail root on the 7th day after primary immunization	*In vivo*: 0.63, 1.26, 2.52 cm^2^ per ankle per day for 7 days, 4 h per day; *In vitro*: 12.5, 25, 50 μM (SZAP skin-permeable component mixture formulated according to MS results based on vanillin content)	① ↓ Arthritis scores and plantar thickness; ② ↓ Pathological lesions such as synovial hyperplasia and vascularisation; ③ ↓ Spleen/thymus index; ④ ↓ Serum IL-6/VEGF/TNF-α levels; ⑤ ↓ LPS-induced NO release from RAW264.7 cells; ⑥ ↓ p-AKT/p-mTOR/HIF-1α protein expression	Block the AKT/mTOR/HIF-1α signaling pathway, and reduce the production of inflammatory factors (IL-6, TNF-α) and angiogenic factors (VEGF)	C+	Lóp et al. (2022)
Yu-Xue-Bi capsule	Olibanum, Myrrha, Clematidis Radix et Rhizoma, Carthami Flos, Salviae Miltiorrhizae Radix et Rhizoma, Cyathulae Radix, Chuanxiong Rhizoma, Angelicae Sinensis Radix, Curcumae Longae Rhizoma, Cyperi Rhizoma, Astragali Radix	Boswellia neglecta S. Moore, Commiphora myrrha (T. Nees) Engl., Clematis terniflora var. Mandshurica (Rupr.) Ohwi, Carthamus tinctorius L., Salvia miltiorrhiza Bunge, Cyathula officinalis K. C. Kuan, Conioselinum anthriscoides ‘Chuanxiong', Angelica sinensis (Oliv.) Diels, Curcuma kwangsiensis S. G. Lee and C. F. Liang, Cyperus rotundus L., Astragalus mongholicus Bunge	2,5-furandiol, Boscartol G and Przewaquinone A, Methyl tanshinonate, Methylnissolin-3-O-glucoside, 3-Hydroxy-4-methoxybenzoic acid, l-arginine and l-homoarginine	Male Lewis rats with CIA-BS	Primary and secondary immunization with BCII combined with CFA; subcutaneous injection of 0.1 mg/kg adrenaline hydrochloride	100, 200, 400 mg/kg/day for 30 days	① ↓ Arthritis scores and foot swelling; ② ↓ Synovial pathological damage; ③ ↑ BV/TV, BMD; ④ Improvement in indicators of blood stasis syndrome; ⑤ Alleviation of mechanical/cold hyperalgesia; ⑥ ↓ VEGF/HIF-1α/iNOS, ↑ eNOS; ⑦ ↓ SDHA/SUCN/TNF-α/IL-1β, ↑ SDHB; ⑧ ↓ TRPV1/TRPA1/SUCNR1 protein expression	Inhibit the SUCNR1/HIF-1α/TRPV1 axis, reduce SDHA-mediated succinate accumulation, block the HIF-1α/VEGF angiogenic pathway, restore the balance between eNOS and iNOS, and alleviate inflammation and pain	A^+^	Willson et al. (2022)

IFA: Incomplete Freund’s adjuvant; CFA: Complete Freund’s adjuvant; BCII: Bovine type II, collagen; CCII: Chicken type II, collagen; BS: Blood Stasis; FLS: Fibroblast-like synoviocytes; HUVEC: Human Umbilical Vein Endothelial Cells; AIA: Adjuvant-Induced Arthritis; POWO: Plants of the World Online (http://www.plantsoftheworldonline.org); MPNS: Medicinal Plant Names Services (http://mpns.kew.org/mpns-portal/).

**TABLE 5:** Preclinical studies on monomeric natural compounds targeting HIF-1α-mediated angiogenesis in the treatment of rheumatoid arthritis.

Natural compound	Botanical source	Model/Cell	Induction method	Range of dosage	Key effects	Molecular mechanism	Evidence level	References
α-Mangostin	Garcinia mangostana L	*In vivo*: Male Wistar rats with AIA *In vitro*: HUVEC	Intradermal injection of 0.1 mL CFA containing BCG into the left hind paw	*In vivo*: 30 mg/kg via intragastric administration (microemulsion) *In vitro*: 4 μg/mL	① ↓ Arthritis score and foot swelling; ② ↓ Inflammatory cell count; ③ ↓ HIF-1α/VEGF/IL-6/TGF-β; ④ ↓ Matrigel-embedded angiogenesis; ⑤ ↓ HUVEC migration and tubule formation; ⑥ ↓ Endothelial sprouting in aortic rings; ⑦ ↓ NOX/MDA, ↑ GSH/SOD; ⑧ Regulation of glucose metabolism and restoration of LDH activity	Inhibit aerobic glycolysis via suppressing LDH, and downregulate the HIF-1α/VEGF signaling pathway	B+	Reichard and Asosingh (2019)
Clematichinenoside AR	Clerodendrum chinense (Osbeck) Mabb	*In vivo*: Male SD rats with CIA *In vitro*: RA-FLS, HUVEC	Primary immunization: Intradermal injection of 0.1 mL CCII combined with CFA at tail base and right hind paw Secondary immunization: Same treatment as primary immunization on day 7 after initial immunization	*In vivo*: 8, 16, 32 g/kg/day for 14 days *In vitro*: 300, 400, 500 nmol/L	① ↓ Arthritis scores and foot swelling; ② ↓ Spleen/thymus index; ③ ↓ Pathological joint damage; ④ ↓ Synovial CD31/CD34 expression; ⑤ ↓ Immature blood vessels (CD31^+^/αSMA^−^); ⑥ ↓ Proliferation, migration and invasion of RA-FLS, ↑ apoptosis; ⑦ ↓ HUVEC tubule formation	Directly bind to HIF-1α with high affinity inhibit HIF-1α/VEGFA/ANG2 axis to suppress synovial angiogenesis	D+	Chang et al. (2014)
Total saponins of Panax japonicus	Panax japonicus (T.Nees) C.A.Mey	*In vivo*: Male DBA/1J mice with CIA *In vitro*: EA.hy926 cells	Primary immunization: BCII combined with CFA Secondary immunization: CII combined with IFA on day 14 after primary immunization	*In vivo*: 30, 150 mg/kg/day for 11 days *In vitro*: 50, 100, 200 μg/mL	↓ Arthritis index, hind paw thickness and number of swollen joints; ↓ Synovial pathological damage; ↓ CD31^+^ expression and vascular density in the synovium; ↓ serum and synovial protein levels of HIF-1α, VEGFA and ANG-1; ↓ splenic mRNA expression of HIF-1α, VEGFA and ANG-1; ↓ IL-6-induced tubule formation in EA.hy926 cells; ↑ thermal stability of HIF-1α, VEGFA and ANG-1 proteins	Target the HIF-1α/VEGF/ANG-1 axis to inhibit pathological angiogenesis	C+	Tong et al. (2025)
Matrine	Sophora flavescens Aiton	*In vivo*: Male SD rats with CIA *In vitro*: RA-FLS, HUVEC	Primary immunization: Subcutaneous injection of 1 mL BCII + IFA at tail base Secondary immunization: Subcutaneous injection of 0.5 mL BCII + IFA at tail base on day 7 after primary immunization	*In vivo*: 100 mg/kg/day for 28 days *In vitro*: 0.5, 1.0, 1.5, 2.0 mg/mL	↓Arthritis scores and foot swelling; ↓pathological damage such as synovial angiogenesis; ↓ Expression of IL-1β, IFN-γ, VEGF, PLGF and HIF-1α in ankle joint tissue; ↓ Ang-1/2 protein levels; ↓ IL-1β-induced proliferation of RA-FLS; ↓ VEGF165-induced proliferation of HUVEC; ↓ Migration of RA-FLS; ↓ HUVEC tubule formation and number of branches	Inhibition of p-Akt downregulates the HIF-VEGF-Ang axis and suppresses the PI3K/Akt pathway, thereby inhibiting synovial angiogenesis	D+	Pei-rong et al. (2021)
Sinomenine	Sinomenium acutum (Thunb.) Rehder and E.H.Wilson	*In vivo*: C57BL/6J mice with CIA	Primary immunization: Intradermal injection of 0.1 mL BCII + CFA at tail base Secondary immunization: Intradermal injection of 0.1 mL BCII + IFA at tail base	30, 100, 300 mg/kg/day for 11 days	① ↓ Arthritis index; ② ↓ Pathological damage to the synovium; ③ ↓ Density of CD31^+^ cells and microvessels in the synovium; ④ ↓ Expression of HIF-1α/VEGF/ANG-1	Suppress the HIF-1α-VEGF-ANG-1 signaling axis	D^-^	Pan et al. (2025)
Resveratrol	Veratrum album L	*In vivo*: Female SD rats with CIA *In vitro*: RSC-364 cells	Primary immunization: Subcutaneous injection of 0.1 mL BCII + IFA at tail base Secondary immunization: Same treatment on day 7 after primary immunization	*In vivo*: 200, 400 mg/kg/day for 21 days *In vitro*: 25, 50 μmol/L	① ↓ Arthritis score; ② ↓ Synovial pathological damage; ③ ↓ Serum MDA, ↑ SOD activity; ④ ↓ IL-1β/MCP-1/IL-6/TNF-α; ⑤ ↓ RSC-364 cell proliferation, induction of apoptosis	Inhibiting the p38/JNK-HIF-1α-ROS-inflammation axis exerts anti-inflammatory and anti-angiogenic effects	D+	Zhang et al. (2025a)
Coix Seed Oil	Coix lacryma-jobi L	*In vivo*: Female SD rats with CIA *In vitro*: FLS	Primary immunization: Subcutaneous injection of CCII + CFA at tail base Secondary immunization: Same treatment on day 7 after primary immunization	*In vivo*: 2.1, 4.2, 8.4 g/kg/day for 28 days *In vitro*: 500 μg/mL	① ↓ Arthritis index and foot swelling; ② ↓ Pathological damage to joints and synovium; ③ ↑ Micro-CT bone parameters; ④ ↓ Serum HIF-1α/VEGF-A; ⑤ ↓ Immature blood vessels in the synovium; ⑥ ↓ FLS migration; ⑦ ↓ Microvascularisation in the aortic ring; ⑧ ↑ SIRT1, ↓ HIF-1α/VEGF-A/CD31; ⑨ Inhibition of HIF-1α nuclear translocation	Upregulate SIRT1 expression to inhibit HIF-1α/VEGF-A pathway and further suppress synovial angiogenesis	D+	Xu et al. (2024)
Baicalein	Scutellaria baicalensis Georgi	*In vivo*: Female SD rats with CIA	Primary immunization: Subcutaneous injection of 0.1 mL BCII + CFA into right hind paw Secondary immunization: Subcutaneous injection of 0.1 mL BCII + CFA at tail base on day 7 after primary immunization	Intragastric administration: 10, 30 mg/kg/day for 28 days	① ↓ Arthritis indices and toe swelling; ② ↓ Thymus/spleen indices; ③ ↓ Synovial pathological damage; ④ ↓ Synovial TNF-α/IL-6; ⑤ ↓ VEGF/KDR expression; ⑥ ↓ HIF-1α/VEGF protein expression	Block HIF-1α/VEGF pathway to relieve inflammatory response and pathological angiogenesis	D+	Chen et al. (2015)
Artesunate	Artemisia annua L	RA-FLS	Stimulation with 10 ng/mL TNF-α and hypoxia (1% O_2_)	10 μmol/L	① ↓VEGF/IL-8 secretion in RA-FLS; ② ↓nuclear expression and translocation of HIF-1α; ③ ↓TNF-α/hypoxia-induced Akt phosphorylation	Inhibit PI3K/Akt pathway to downregulate HIF-1α expression and reduce secretion of VEGF and IL-8	A^-^	Tao et al. (2016)

The data in the ‘Source’ column of the table is sourced from the original literature and has been listed following verification via (POWO(PPOWO: Plants of the World Online (http://www.plantsoftheworldonline.org); MPNS: Medicinal Plant Names Services (http://mpns.kew.org/mpns-portal/).

### 4.1 Natural medicine formulations

**FIGURE 3:**
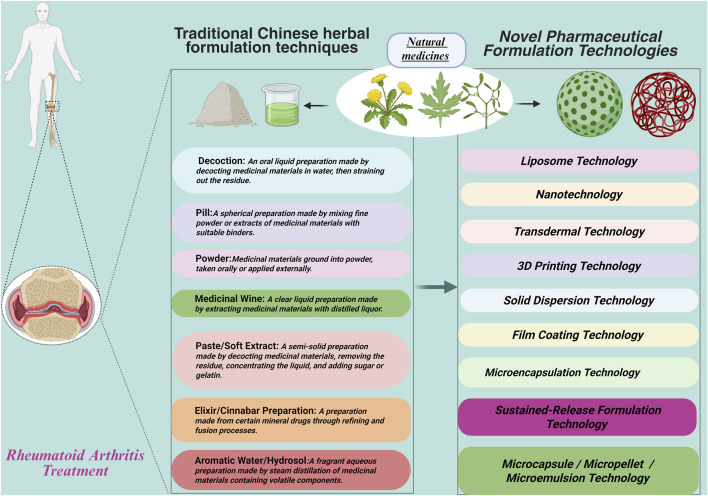
Transition in natural product delivery for RA therapy. The schematic diagram illustrates the transition from traditional oral/topical formulations (decoctions, plasters, and pills) to modern nano-delivery systems (HIF-1α siRNA nanoparticles, biomimetic liposomes, and hydrogel-nanocomposites). The innovative systems achieve efficient disruption of the “hypoxia-HIF-1α-angiogenesis” vicious cycle in RA synovium through a three-tier mechanism (Díaz-González and Hernández-Hernández, 2023): inflammatory joint-targeted accumulation (Radu and Bungau, 2021), microenvironment-responsive release, and (Elshabrawy et al., 2015) precise intervention of the HIF-1α pathway, thereby overcoming the limitations of traditional formulations, including low bioavailability, poor targeting, and rapid metabolism [Created with BioRender (https://app.biorender.com/)].

Natural medicines exhibit the characteristics of multi-component synergistic effects and multi-target actions. Through the synergistic effects of multiple components, countless CM compound formulations composed of different natural medicines can effectively treat refractory diseases (Tong et al., 2025; Chen Ruilian et al., 2025). Meanwhile, due to their low cost and high safety, they have attracted increasing attention. Based on the clinical syndrome classification of RA, CM formulations have demonstrated significant clinical efficacy in improving the symptoms of this disease. Classic formulations such as Wutou decoction, Fangji Huangqi Decoction have been used for RA treatment for over 1,000 years (Liu et al., 2025; Pan et al., 2025; Zhang Y. et al., 2025). Wutou decoction can inhibit the formation of pannus in the synovial tissue of RA. Studies have shown that Wutou decoction can reduce the production of CD31^+^/αSMA^−^ and the total vascular concentration in the synovial membrane of joints, and dose-dependently inhibit the migration, invasion, adhesion, and lumen formation of HUVEC induced by VEGF or Human Synovial Fibroblast Cell Line MH7A (MH7A), as well as the apoptosis of MH7A cells (He et al., 2018). Its mechanism primarily involves inhibiting PI3K-AKT-mTOR, the key signaling pathway, to reduce the protein synthesis and accumulation of HIF-1α, subsequently downregulating the expression of VEGF, ANG1, and their receptors, and inhibiting synovial hyperplasia and angiogenesis (Ba et al., 2021). Another classic formulation, Fangji Huangqi Decoction, is a renowned CM formulation with outstanding efficacy in treating RA of wind-cold-dampness or wind-dampness type (Jiang et al., 2022). Studies have demonstrated that Fangji Huangqi Decoction possesses anti-inflammatory and antioxidant properties so that it can significantly alleviate arthritis symptoms in CIA model rats, inhibit synovial angiogenesis, suppress the expression of CD31, HIF-1α, VEGF, and waveform protein in synovial tissue, and regulate the balance of autophagy and apoptosis in FLS. By downregulating the expression of HIF-1α and its downstream factor VEGF, it inhibits synovial angiogenesis and reduces the angiogenic capacity of HUVEC induced by MH7A cells, which suggests that its mechanism involves intervention in the “synovial cell-endothelial cell” crosstalk (Xu et al., 2024). Huayu Tongbi Formula is a clinically effective CM metabolite formulation for RA treatment (Chen et al., 2015). Studies indicate that this formulation contains blood-activating agents and stasis-resolving agents with angiogenic inhibitory effects, making it a promising therapeutic option (Tao et al., 2016) through the way of directly targeting the HIF1A-VEGFA-ANGPT angiogenic core axis. *In vitro* experiments have demonstrated its efficacy in inhibiting cell-cell crosstalk between RA FLS and HUVEC, reversing key angiogenic steps such as cell proliferation, migration, adhesion, and lumen formation, and significantly reducing serum levels of VEGFA, ANGPT1, and ANGPT2 (Chen Z. et al., 2025). Research on the inhibition of the AKT/mTOR pathway in the upstream signaling of HIF-1α has also revealed that Shexiang Zhui Feng Analgesic Plaster can inhibit the AKT/mTOR/HIF-1α pathway and reduce the release of key inflammatory mediators such as IL-6, VEGF, and TNF-α, achieving dual anti-angiogenic and anti-inflammatory effects, which provides a basis for external anti-angiogenic therapy (Wang et al., 2023). The Chinese patented botanical drugs Yu-Xue-Bi Capsule has been widely used to treat RA with blood stasis syndrome, with satisfactory clinical efficacy (Geng et al., 2022). By modulating the SUCNR1/HIF-1α/TRPV1 axis, Yu-Xue-Bi Capsule inhibits the HIF-1α/VEGF signaling pathway and protects vascular function by increasing the eNOS/iNOS ratio, targeting metabolic sensing and improving endothelial tolerance, which provides a novel approach for treating RA with blood stasis syndrome (Chen W. et al., 2025) (Figure 3).

### 4.2 Natural active metabolites

The monomeric metabolites derived from traditional Chinese medicine, with their well-defined components and clear target points, precisely target HIF-1α-related pathways, facilitating precise mechanism studies and serving as an important source for discovering lead metabolites targeting HIF-1α. α-Mangostin (MAN), derived from Garcinia mangostana, has been shown in studies on the mechanism of RA that MAN can intervene in HIF-1α at the metabolic level by inhibiting aerobic glycolysis in AIA rats and FLS, alleviating inflammation-related hypoxia, thereby reducing the stability of HIF-1α and antagonizing VEGF-induced pathological neovascularization in RA (Jiang et al., 2021). Clematichinenoside AR (CAR) has been confirmed as a direct inhibitor of HIF-1α, which significantly reduces angiogenesis and synovial pathological changes in CIA rats by inhibiting the HIF-1α/VEGFA/ANG2 axis, and suppresses the proliferation, migration, and neointimal formation of RA FLS and HUVEC. Pathological staining showed no significant abnormalities in major organs of rats after intervention with a CAR dose of 32 g/kg, indicating no obvious toxic effects of CAR (Yuan et al., 2025). Panax japonicus, widely planted in the southwestern regions of China and known as Zhujieshen in Chinese, exhibits analgesic, anti-inflammatory, antioxidant, and joint swelling-relieving effects. Its main active component, Total Saponins of Panax japonicus (TSPJ), is widely used in the treatment of RA. Studies have shown that anti-angiogenesis is an effective strategy of TSPJ against RA. Network pharmacology and bioinformatics initially reveal that TSPJ is the key target in VEGF and HIF-1 signaling pathways. Subsequent *in vitro* and *in vivo* experiments also demonstrate that it can inhibit joint swelling and angiogenesis in CIA mice, with efficacy comparable to Lificiguat, the HIF-1α inhibitor. So that can reduce the expression of HIF-1α, VEGF, and ANG-1 in rat synovial tissue. *In vitro* experiments indicatethat it can inhibit the tube formation of EA. hy 926 cells, and its anti-angiogenic effect may be related to the inhibition of the HIF-1α/VEGF/ANG-1 axis (Guo et al., 2024). Matrine (Mat), an extract of Sophora flavescens, inhibits the proliferation, migration, and lumen formation of FLS and HUVEC in anti-RA studies. Thereby accelarating the apoptosis of FLS in CIA rats. Mechanistic studies reveal that Mat alleviates angiogenesis in CIA rats by inhibiting the PI3K/Akt signaling pathway and the HIF-VEGF-ANG angiogenesis axis (Ao et al., 2022), thereby relieving RA symptoms. Sinomenine (SIN) is an alkaloid extracted from the medicinal plant Sinomenium acutum, which has been used in China for over 2,000 years to treat RA. It has been demonstrated in experimental animal models and clinical applications to have potential anti-inflammatory, analgesic, immunosuppressive, and anti-angiogenic effects during the treatment of immune-related diseases (Kok et al., 2005). SIN significantly reduces the severity of arthritis and microvascular density in CIA mice, inhibiting angiogenesis to alleviate CIA. Its mechanism of action involves anti-angiogenic effects through the HIF-1α-VEGF-ANG-1 axis (Feng et al., 2019). Resveratrol, a non-flavonoid polyphenolic organic metabolite derived from Veratrum, has been shown to improve bovine type II collagen-induced synovitis in rat models and rheumatoid arthritis-related pathological features such as inflammatory cell infiltrati and angiogenesis in synovial tissues, while eliminating reactive oxygen species (ROS) and inflammation. Further studies have found that RSV downregulates the level of HIF-1α in RA FLS stimulated by IL-1β, reduces ROS accumulation to suppress inflammatory responses and cell proliferation, and induces synovial cell apoptosis, while also attenuating HIF-1α-mediated angiogenesis (Yang et al., 2018). Other studies have shown that RSV inhibits angiogenesis through the STAT3/HIF-1/VEGF pathway, exerting its anti-RA function (Nazari-Khanamiri and Ghasemnejad-Berenji, 2022). Coix Seed Oil (CSO), derived from the fruit of Coix lacryma-jobi, has been shown to inhibit the secretion of pro-inflammatory cytokines in macrophages by suppressing the HIF-1α/STAT3/NLRP3 pathway, thereby slowing the progression of joint inflammation in CIA rats (Wu et al., 2023). Further studies indicate that CSO reduces the levels of HIF-1α and VEGFA proteins in both CIA rats and fibroblast-like cells (FLS), alleviates the histopathological deterioration of synovial and joint tissues, inhibits CD31+/αSMA−-marked immature blood vessels in the synovium, and decreases VEGF-induced microvessels in the aortic ring. Its core mechanism involves Coix Seed Oil exerting anti-arthritis effects by modulating the SIRT1/HIF-1α/VEGFA signaling pathway, which reduces immature synovial angiogenesis and fibroblast-like synovial cell migration (Xu et al., 2023). Baicalein (BA) inhibits inflammatory responses and pathological angiogenesis in RA rats by regulating the HIF-1α/VEGF signaling pathway, thereby alleviating synovial tissue injury (Du et al., 2022). Artemisinin derivatives, particularly artesunate, inhibit PI3 kinase/Akt activation, which may suppress HIF-1α expression and hypoxia-induced VEGF and IL-8 secretion to inhibit angiogenesis for RA treatment (He et al., 2011) (Figure 2).

## 5 Innovative strategies using natural metabolites to intervene in HIF-1α-angiogenesis for the treatment of RA

### 5.1 Advantages of combined therapeutic interventions of natural medicine for the treatment of RA

RA is a progressive inflammatory autoimmune disease dependent on angiogenesis, and current RA botanical drugs therapies are closely associated with anti-angiogenic mechanisms. The primary medications for RA treatment are nonsteroidal anti-inflammatory botanical drugs (NSAIDs) and disease-modifying anti-rheumatic botanical drugs (DMARDs), which may influence synovial angiogenesis through various mechanisms. NSAIDs such as indomethacin, celecoxib, and rofecoxib can inhibit RA angiogenesis via different mechanisms (Fidahic et al., 2017), while DMARDs like methotrexate (MTX) and leflunomide partially block angiogenesis by suppressing VEC proliferation (Nabai et al., 2015). Among these, MTX serves as a positive control botanical drugs in RA *in vitro* and *in vivo* studies investigating HIF-mediated angiogenesis. Additionally, biological inhibitors such as infliximab and cyclosporine have been shown to reduce RA angiogenesis by inhibiting HIF-downstream VEGF expression (Liu et al., 2013; Scott, 2017). The combination of TNF-α monoclonal antibodies and MTX significantly inhibits synovial pannus formation (Wang et al., 2021). However, these therapies are often accompanied by adverse effects such as hepatotoxicity, nephrotoxicity, bone marrow suppression, and gastrointestinal discomfort (Wang et al., 2018). In contrast, natural botanical drugs, including bioactive metabolites, extracts, and formulation botanical drugs, have emerged as promising therapeutic alternatives for rheumatoid arthritis in modern society. In recent years, combination therapies have become a promising treatment approach, making the integration of natural metabolites with traditional DMARDs and biologics an attractive strategy for synergistic efficacy and reduced toxicity. A preliminary clinical study on Huayu Tongbi formula has revealed that its combination with MTX demonstrates efficacy comparable to LEF + MTX in improving laboratory-related indicators and disease activity score 28 (DAS28), with fewer adverse reactions (Chen et al., 2015). Additionally, multiple clinical studies on Wutou decoction have shown that incorporating WTD into clinical practice can effectively alleviate clinical symptoms in RA patients, reduce laboratory-related indicators, and decrease the dosage of NSAIDs and DMARDs, thereby preventing or mitigating adverse effects of potentially toxic botanical drugs such as nonsteroidal anti-inflammatory botanical drugs, glucocorticoids, and opioids in RA patients, making it a safe and effective treatment (Liu et al., 2025). The Chinese medicine Tripterygium wilfordii Hook F (TwHF), used for treating joint pain, fever, chills, edema, and local inflammation, has been approved and routinely applied in RA treatment. It has been demonstrated in a 24-week randomized controlled trial that monotherapy with TwHF is non-inferior to MTX monotherapy in controlling disease activity in active RA patients, and TwHF combined with MTX show superior efficacy compared to MTX monotherapy (Lv et al., 2015). Although natural medicines have garnered widespread attention due to their therapeutic potential in regulating HIF-1α-mediated angiogenesis for RA treatment, their quality control and safety issues still remain a matter of concern. Natural medicine formulations are characterized by complex active metabolites, whose quality are affected by factors such as plant species, soil, and climate. Moreover, some important metabolite formulations may contain components potentially detrimental to human health. For instance, ephedrine, one of the most critical active components in Ephedra sinica, a kind of natural medicine inWutou Decoction, along with other alkaloids such as benzoic aconitine, benzoic neaconitine, and benzoic aconitine, exhibit significantly higher total content in model rats than in healthy rats, yet these components may serve as key pharmacological substances exerting direct effects at target sites (Liu et al., 2025). Therefore, clinical monitoring and pharmacokinetic studies are crucial before recommending combination therapies.

### 5.2 Enhancement strategies for natural medicine intervention in HIF-1α-mediated angiogenesis for RA

**FIGURE 4:**
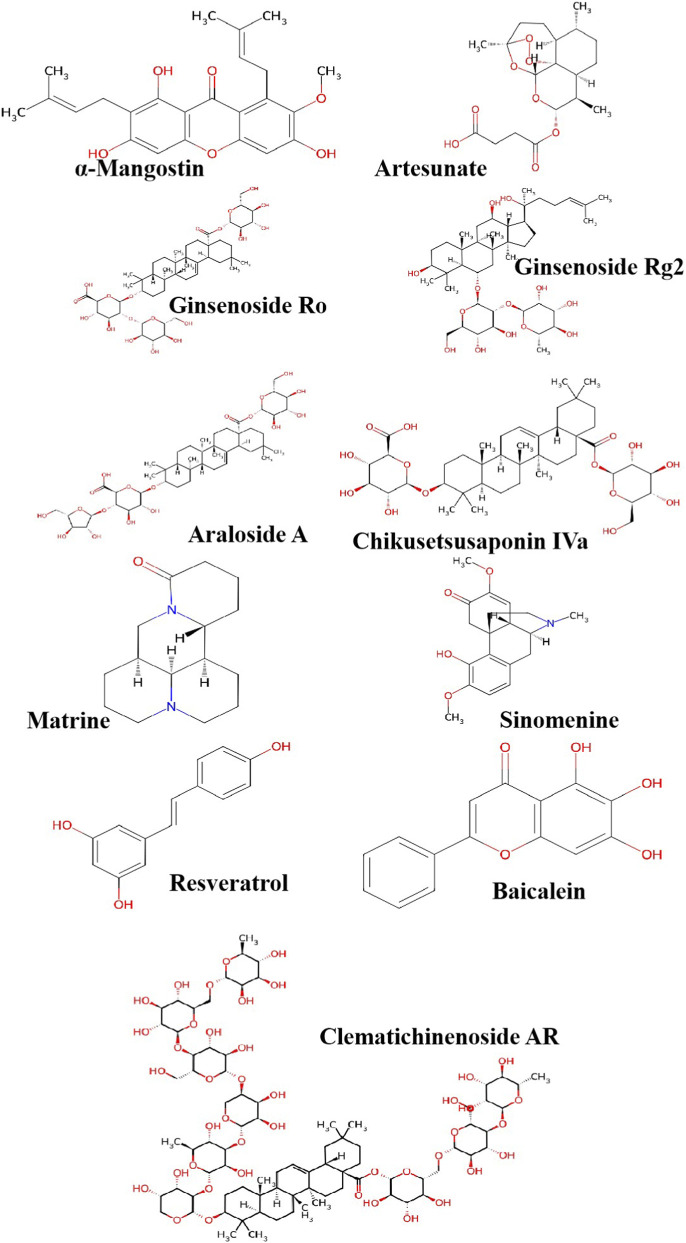
Chemical structure of Natural active metabolites, from (https://www.chemspider.com/).

HIF-1 plays a crucial role in RA angiogenesis, and targeting HIF-1α-mediated vascular formation represents a promising therapeutic approach for RA. In recent years, scientific research on natural medicines has made substantial progress, particularly in elucidating material bases, mechanisms, formulation rationality, and quality standards. However, significant challenges still persist in the development of natural medicines. Current investigations of natural medicines predominantly focus on metabolite formulas or crude extracts. Traditional formulations such as decoctions, plasters, and pills generally exhibit low bioavailability, poor targeting, rapid metabolism, and short therapeutic half-lives. The active metabolites responsible for the botanical drugs’s efficacy and their specific molecular targets on HIF-1α remain unclear, constraining their precision therapeutic applications and clinical translation. Insufficient botanical drugs retention at lesion sites and non-specific tissue distribution reduce therapeutic efficacy and increase risks of systemic immunosuppression-related infections. In recent years, advances in botanical drugs delivery technologies, particularly nanostructure-based and scaffold-assisted delivery platforms, have enhanced botanical drugs accumulation at target sites, controlled botanical drugs release, prolonged therapeutic duration, reduced dosage and administration frequency, and ultimately improved treatment outcomes. These delivery systems selectively accumulate at synovial inflammatory sites, increase local botanical drugs concentrations, significantly enhance the inhibition efficiency of HIF-1α in synovial FLS and VEC, and minimize damage to healthy tissues, thereby providing novel strategies for precision treatment of RA (An et al., 2024; Liu et al., 2022; Yan et al., 2025). Beyond nanocarrier optimization, innovative botanical drugs delivery approaches have fundamentally transformed RA therapeutic paradigms. Currently, scaffold-based delivery systems including hydrogels, microneedles, and preformed solid implants enable effective intra-articular botanical drugs loading, achieving sustained and controlled botanical drugs release with efficient enrichment at synovial lesion sites (Zhang J. et al., 2025). Preclinical *in vitro* studies demonstrate that a novel TP-PLGA-Au@RGD/HA hydrogel incorporating triptolide significantly reduces the migratory and invasive capacities of RA FLS and ameliorates inflammatory conditions, maximizing therapeutic efficacy while minimizing dose-related adverse effects (Li et al., 2022). Furthermore, biomimetic nanoliposomes (FMPlipo@C) utilizes homologous targeting capabilities to precisely deliver active components to synovial cells *in vivo*. It effectively protects cartilage and attenuates bone erosion through HIF-1α/iNOS/NLRP3 axis inhibition (Chen M. et al., 2025). These investigations provide valuable insights for developing innovative RA therapeutic strategies and establish a promising pathway for precision treatment of RA by targeting HIF-1α-mediated angiogenesis with natural medicines (Figure 4).

## 6 Conclusions and perspective

HIF-1α, as the core hub connecting the hypoxic microenvironment of RA synovium with pathological angiogenesis, has become a critical therapeutic target for preventing synovial pannus and joint damage in RA. Natural medicines (including natural medicine formulations and natural active metabolites) demonstrate unique value in anti-RA angiogenesis. Through existing research, we have found that the majority of current studies are preclinical in nature. However, these preclinical studies suffer from key methodological limitations. At the level of *in vivo* animal experiments, the diversity of animal models is limited (primarily restricted to CIA/AIA models), coupled with a dearth of disease-syndrome integrated models that reflect different clinical phenotypes, disease stages, and comorbidities. Furthermore, there is a significant lack of standardisation regarding key variables in experimental protocols, such as animal strain, sex, modelling methods and immunisation protocols, resulting in considerable heterogeneity; Research on natural medicine formulations largely relies on traditional usage and lacks modern pharmacokinetic parameters. Studies on individual compounds exhibit wide variations in dosage. Most individual compounds have low oral bioavailability, and there is a significant difference in magnitude between *in vitro* effective concentrations and *in vivo* plasma drug concentrations, with no standardized protocols yet established. In intervention studies involving traditional Chinese medicine (TCM) formulations, although some formulations state that their moderate doses are derived from clinically equivalent doses, standardized operating procedures are still lacking for the preparation of drug-containing serum used in vitro experiments. Currently, no clear dose-response relationship has been established for the animal dosing regimens used to prepare drug-containing serum. Dosages are typically four or five times the clinical dose or are administered as freeze-dried powder, whereas the maximum dosage used in vivo animal experiments is no more than twice the clinical dose, making it difficult to establish a reasonable correlation between the two. Furthermore, research on TCM formulations often remains limited to the enumeration of bioavailable components, failing to elucidate the core pharmacologically active substances or the synergistic mechanisms of multiple components. Moreover, most single natural products lack evidence of direct target binding or verification of selectivity. Particularly noteworthy is the severe inadequacy of safety evaluations, as a large number of toxic TCM formulations and natural products have not undergone systematic organ toxicity testing or long-term safety margin analysis. At the mechanistic level, a large number of studies rely on computational biology or literature mining and target prediction, leading to an excessive focus on known pathways such as HIF-1α, VEGF, PI3K/AKT, and mTOR. Moreover, RA is characterized not only by joint destruction but also by significant chronic pain and central sensitization. Although “vascular-bone coupling” and “vascular-neural coupling” are among the core pathological mechanisms by which pathological angiogenesis leads to joint destruction and pain, most current research focuses solely on the angiogenesis they mediate, while insufficient attention has been paid to exploring the pathological changes (e.g., “vascular-bone coupling” or “vascular-neural coupling”) induced by this angiogenesis. Furthermore, the predominantly preclinical nature of current mechanistic research, coupled with a lack of clinically relevant studies, has thereby limited the development of herbal formulations designed to intervene in HIF-1α-mediated angiogenesis. With deepening understanding of the HIF family, the role of HIF-2α in cartilage destruction and the maintenance of chronic inflammation in RA has gradually come to the fore. Furthermore, given the high homology between HIF-1α and HIF-2α in their DNA-binding domains, both can recognize similar hypoxia-responsive elements, suggesting that the upregulation of VEGF in the complex synovial microenvironment of advanced RA may result from synergistic action between HIF-1α and HIF-2α. Additionally,the modified GRADE framework adopted in this review provides a structured pathway for integrating heterogeneous evidence sources, including clinical samples, animal/cell experiments, computational biology, and literature reviews. Despite this, the method has several methodological limitations and requires critical scrutiny, not least because replacing the traditional “study design” classification with “evidence source grade” may introduce new biases. Clinical studies are often constrained by small sample sizes, lack of control groups, and inadequate adjustment for confounding factors, whereas rigorously designed animal experiments can offer more stringent experimental conditions and causal inferences. Therefore, the current grading system carries a systematic risk of overestimating the weight of clinical evidence while underestimating the contribution of high-quality preclinical research. Second, the PAINS rule was originally developed for medicinal chemistry and high-throughput screening; however, when applied to plant-derived polyphenols, quinones, and alkaloids, it may generate false-positive alerts. Notably, although this review adopted a more cautious strategy by adjusting the grade based on whether the PAINS compound was a core active metabolite, such subjective judgments inevitably lead to inconsistencies in scoring across different literature sources.

In consideration of the above limitations, we propose the following specific and actionable directions for future research: establishing uniform experimental standards for animal models, including clarifying strain, sex, modeling methods, and immunization protocols, and developing disease-syndrome integrated models; identifying active metabolites, establishing a standardized quality control system, and systematically conducting dose-response studies, pharmacokinetic analyses, and comprehensive safety evaluations; and establishing a more comprehensive research quality rating system to better assess research quality and promote high-quality development in the field. Meanwhile, targeted delivery systems based on innovative technologies such as nanodelivery, hydrogels, and microneedles should be developed. Dual-target synergistic inhibition strategies targeting both HIF-1α and HIF-2α, as well as biomarker-guided precision therapy, warrant exploration. In-depth research should also be conducted into mechanisms such as “vascular-bone coupling” and “vascular-neural coupling” induced by the HIF-1α/VEGF axis to elucidate the underlying pathological mechanisms of bone destruction and chronic pain in rheumatoid arthritis. At the same time, future therapeutic strategies should not be limited to the single target HIF-1α but should explore “integrated” intervention approaches targeting the entire HIF family, in order to simultaneously block HIF-1α-mediated angiogenesis and HIF-2α-mediated cartilage degradation. Crucially, future research should strive toward clinical translation; however, achieving this goal will require the application of modern scientific and technological methods to address core issues such as chemical complexity, pharmacokinetics, safety assessment, and evidence of efficacy. Only then can the wisdom of TCM be transformed into a new treatment option for RA recognized by modern evidence-based medicine.

## 7 Conclusion

As a central hub linking the hypoxic synovial microenvironment to pathological angiogenesis in RA, HIF-1α represents a highly promising therapeutic target. Accumulating preclinical evidence has demonstrated that both Chinese medicine formulations and natural metabolite-derived single agents can inhibit synovial angiogenesis by modulating the HIF-1α signaling network through multiple pathways, positioning them as encouraging candidate drugs for further development. Nevertheless, the vast majority of available evidence remains at the preclinical stage. The clinical translation of natural medicines for RA treatment faces substantial challenges, including issues related to standardization, safety, and poorly defined pharmacokinetic profiles, coupled with a paucity of high-quality clinical trials. Consequently, rigorous validation in clinical settings is urgently required.
